# Supplementary material for: Modeling glioblastoma heterogeneity as a dynamic network of cell states
Source: Mol Syst Biol. 2021 Sep 16;17(9):e10105. doi: 10.15252/msb.202010105 (PMC8444284; doi:10.15252/msb.202010105)
Supplement: Supplementary file 6 — Source Data for Figure 5 [file MSB-17-e10105-s004.zip › Figure5A_sourcedata/GSEA_3017/hallmarks_stateE.GseaPreranked.1621934677158/neg_snapshot.html]

Snapshot of 8 enrichment plots

Table: Snapshot of enrichment results

|  |  |  |
| --- | --- | --- |
|  |  |  |
|  |  |  |
|  |  |  |
